# Expression of the epithelial polymeric immunoglobulin receptor is decreased in allergic rhinitis and eosinophilic rhinosinusitis

**DOI:** 10.1186/2045-7022-3-S2-O8

**Published:** 2013-07-16

**Authors:** Cloé Hupin, Philippe Rombaux, Marylène Lecocq, Charles Pilette

**Affiliations:** 1 Cliniques Universitaires Saint-Luc, Service d'Oto-Rhino-Laryngologie, Brussels, Belgium; 2 Université Catholique de Louvain, IREC, Pole de Pneumologie, ORL & Dermatologie, Brussels, Belgium

## Background

Transcytosis of immunoglobulin A (IgA) through polarized bronchial and sinonasal epithelial cells is mediated by the polymeric immunoglobulin receptor (pIgR), which represents the rate-limiting factor for this frontline protective mechanism in the airways. pIgR expression is decreased in COPD, lung cancer and nasopharyngeal carcinoma, while its role in sinonasal chronic inflammatory diseases has not been explored. The aim of this study was thus to assess pIgR expression in sinonasal mucosa of patient with chronic rhinosinusitis with (CRSwNP) or without polyps (CRSsNP) and in allergic rhinitis (AR), as well as IgA and SC (the released soluble part of the pIgR) in nasal secretions.

## Methods

Nasal and ethmoidal biopsies, as well as nasal fluid, were collected from patients with CRSwNP, CRSsNP and AR, as compared to control subjects. pIgR expression was analyzed by RT-qPCR and immunohistochemistry. IgA and SC were measured in nasal secretions by ELISA. Quantification of mucosal eosinophils was performed following hematoxylin-eosin staining.

## Results

RT-qPCR showed a significant reduction of pIgR expression in ethmoidal biopsies from CRSwNP (p = 0,01) and AR (p = 0,04). This reduction was confirmed at the protein level by immunohistochemistry, and resulted into reduced levels of SC and trends for reduced IgA in nasal secretions from these patients. Decreased pIgR expression was mainly observed in patients with increased mucosal eosinophils.

## Conclusion

Epithelial pIgR expression is decreased in patients with CRSwNP and AR, results in decreased SC (and IgA) in nasal secretions, and closely relates to Th2-type eosinophilic inflammation. Whether this defect leads to impaired defense of the upper airways against pathogens remains to explore.

